# Insights into the Human Microbiome and Its Connections with Prostate Cancer

**DOI:** 10.3390/cancers15092539

**Published:** 2023-04-28

**Authors:** Raluca Munteanu, Richard-Ionut Feder, Anca Onaciu, Vlad Cristian Munteanu, Cristina-Adela Iuga, Diana Gulei

**Affiliations:** 1 Department of In Vivo Studies, Research Center for Advanced Medicine-MEDFUTURE, “Iuliu Hatieganu” University of Medicine and Pharmacy, 400337 Cluj-Napoca, Romania; 2 Department of Hematology, “Iuliu Hațieganu” University of Medicine and Pharmacy Cluj-Napoca, Victor Babes Street 8, 400012 Cluj-Napoca, Romania; 3 Department of NanoBioPhysics, Research Center for Advanced Medicine-MEDFUTURE, “Iuliu Hatieganu” University of Medicine and Pharmacy, 400337 Cluj-Napoca, Romania; 4 Department of Pharmaceutical Physics and Biophysics, “Iuliu Hațieganu” University of Medicine and Pharmacy, Louis Pasteur Street 6, 400349 Cluj-Napoca, Romania; 5 Department of Urology, The Oncology Institute “Prof Dr. Ion Chiricuta”, 400015 Cluj-Napoca, Romania; 6 Department of Urology, “Iuliu Hatieganu” University of Medicine and Pharmacy, 400012 Cluj-Napoca, Romania; 7 Department of Proteomics and Metabolomics, Research Center for Advanced Medicine–MEDFUTURE, “Iuliu Hațieganu” University of Medicine and Pharmacy Cluj-Napoca, Louis Pasteur Street 6, 400349 Cluj-Napoca, Romania; 8 Department of Pharmaceutical Analysis, Faculty of Pharmacy, “Iuliu Hațieganu” University of Medicine and Pharmacy, Louis Pasteur Street 6, 400349 Cluj-Napoca, Romania

**Keywords:** prostate cancer, dysbiosis, microbiome as cancer biomarker, bacteria, viruses

## Abstract

**Simple Summary:**

In recent years, numerous research studies have been conducted to investigate the microorganisms encountered in various systems of the human body and their implications for cancer development and progression. This review offers a concise description of the bacteria, fungi, viruses, and other agents that colonize different human body sites and organs together with their involvement in the physiological processes related to prostate carcinogenesis. Moreover, this article provides valuable information regarding the connection between the microbiome and prostate cancer by presenting some therapeutical insights that target the microbiota.

**Abstract:**

The human microbiome represents the diversity of microorganisms that live together at different organ sites, influencing various physiological processes and leading to pathological conditions, even carcinogenesis, in case of a chronic imbalance. Additionally, the link between organ-specific microbiota and cancer has attracted the interest of numerous studies and projects. In this review article, we address the important aspects regarding the role of gut, prostate, urinary and reproductive system, skin, and oral cavity colonizing microorganisms in prostate cancer development. Various bacteria, fungi, virus species, and other relevant agents with major implications in cancer occurrence and progression are also described. Some of them are assessed based on their values of prognostic or diagnostic biomarkers, while others are presented for their anti-cancer properties.

## 1. Introduction

At present, prostate cancer (PCa) is the most common cancer among men. Its causes are largely unknown and its development occurs invisibly because of the lack of specific symptoms that might lead to an early diagnosis. According to the Global Cancer Observatory: Cancer Today, in 2020, PCa was the third most-diagnosed disease and the sixth leading cause of deaths worldwide [1,2].

The prostate-specific antigen (PSA) is the cornerstone of PCa diagnosis at present; however, this lab test often leads to misinterpretations because it presents high sensitivity for the prostate gland but poor specificity for PCa, leading either to the misdiagnosis of the disease or to overdiagnosis and overtreatment [3,4,5]. One of the most common causes of non-malignant increased PSA levels is inflammation, even at a subclinical level [6]. Some researchers assert that inflammation plays a vital function in tumor prostatic development; however, little is known about the triggers that activate or maintain tissue inflammation. One of the causes of tissue inflammation consists of changes in the species of bacteria at the specific site or the installation of the dysregulation in the microbial population within the micro-environment [7,8].

The sum of the microbial entities that co-exist in the human body, represented by bacteria, fungi, archaea, viruses, and protists, form the microbiome of a specific individual, being responsible for complex and niche functions throughout the body [9]. Although this domain is not new, the awareness of the importance of the human microbiome in the prevention, development, and treatment of specific diseases has been noticed only recently [10]. Specifically, numerous cancer research studies have turned to the area of microbiology to investigate how microorganisms in the prostate niche and other areas of the body, such as the gut, may impact the onset, progression, and treatment outcomes of PCa. Incipient studies have focused on diseases, such as colorectal carcinoma, inflammatory bowel disease, obesity, and psoriasis, which seem to have a more obvious link with the microbial environment; however, further data link the state of the microbiota with Pca diagnoses and potential innovative treatments [10].

Specific to Pca, there are two main segments of study with respect to the microbial environment: the direct segment that is represented by the microbial organisms present in the tissue of the prostate or in the urine, and the indirect segment that sums up the other microbiomes within the human body, such as oral and fecal microbiomes [11].

**Figure 1:**
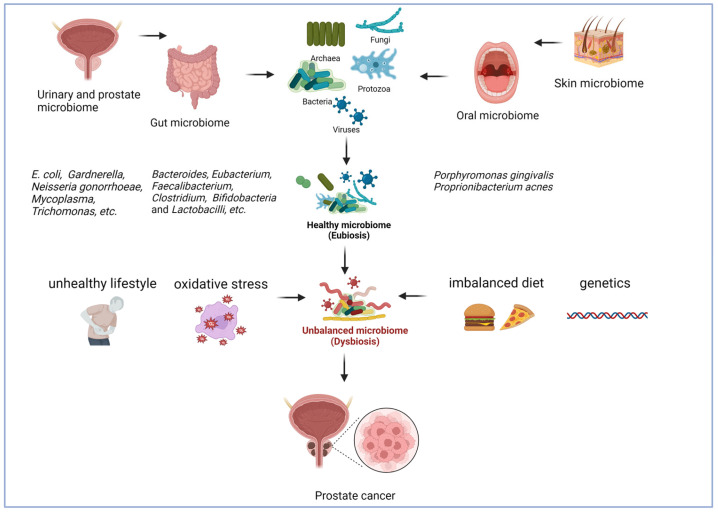
Systems and organs of the human body microbiome have a significant impact on Pca development. The skin, oral cavity, gastrointestinal, urinary, and reproductive systems together with hormonal status are interconnected to maintain the normal physiological state of the human body. Specific microorganisms that colonize different organs have a major impact on the system itself and on the whole organism. Lifestyle, medication, diet, and other factors can lead to an Imbalance in such bacteria, fungi, archaea, protists, and viruses, which can be correlated with disease development, including cancer. The figure was created using BioRender.com.

In this review, we examine the new research on the effects of bacteria colonizing several regions of the human body, including the gastrointestinal tract, the urinary and reproductive systems, the oral cavity, and the skin, upon the installation and development of Pca (Figure 1). Additionally, we explore ways of assessing, preventing, and improving the treatment of Pca by targeting the microbiota.

## 2. Evidence Acquisition

Search strategy: in order to identify the relevant studies, we conducted a systematic search of the PubMed and Web of Science databases using the following search terms: “prostate cancer” AND “microbiota”; “prostate cancer” AND “microbiota” OR “gut microbiota”; “prostate cancer” AND “microbiota” OR “urinary system microbiome”; “prostate cancer” AND “microbiota” OR “prostate microbiome”; and “prostate cancer” AND “microbiota” OR “oral and skin microbiomes”. We restricted our search to studies published in the past 17 years (2005–2022) and only included studies that were written in English.

Inclusion and exclusion criteria: to be included in our review, the studies had to meet the following criteria: (1) the study was published in a peer-reviewed journal; (2) the study investigated the relationship between microbiota and prostate cancer; and (3) the study included human subjects. We excluded studies that were not relevant to our research question or did not meet our inclusion criteria.

Data extraction: we extracted the following data from the selected studies: study design, sample size, age and demographic characteristics of the study population, type of microbiota analyzed, methods used to analyze the microbiota, and the main findings of the study.

Data synthesis: given the heterogeneity of the studies included in our review, we conducted a narrative synthesis of the data. We organized the findings of the studies according to the type of microbiota analyzed (e.g., gut microbiota, urinary microbiota, and prostate microbiota) and the main outcomes investigated (e.g., prostate cancer risk and treatment response).

Limitations: Our review had several limitations that should be considered when interpreting the results. First, our search was limited to studies published in English, which may have led to language bias. Second, the studies included in our review varied in their methods and outcomes, which may have limited the generalizability of our findings. Finally, while we assessed the quality of the selected studies, we did not conduct a formal meta-analysis, which limited our ability to form definitive conclusions about the relationship between microbiota and prostate cancer.

## 3. Gut Microbiome and Pca

As many as ten times more bacteria exist in our bodies as there are human cells, and the vast majority of them reside inside our digestive tracts [12]. Alterations in the microbiome can contribute to tumor formation, especially via the induction of inflammatory responses as a result of antigen stimulation and altered local metabolome. Inflammation is a well-described cancer hallmark, a factor that accelerates the evolution of multiple cancer types [13]. Prostate chronic inflammation, also known as proliferative inflammation atrophy, can occur due to numerous factors, such as bacterial or viral infections, hormonal imbalances, as well as malfunctions of the urinary system [14,15]. The molecular pathways underlying the development of cancer linked with inflammation are complex and include innate and adaptive immune systems. The innate immune system’s activated phagocytic inflammatory cells release highly reactive chemical compounds, such as superoxide, singlet oxygen, hydrogen peroxide, and nitric oxide, which can damage epithelial cells’ DNA through oxidative or nitrosation reactions, or react with other cellular components, such as phospholipids, to cause a free-radical chain reaction. These events result in the destruction of the epithelial cells that are replaced through the division of precursor cells. However, the division that occurs in an environment with DNA-damaging agents can easily generate daughter cells with mutations that can eventually lead to the installation of malignant formations [14].

At present, the research focuses on how alterations in the gastrointestinal and genitourinary microbiota contribute to the development of numerous diseases, including cancer [16]. In terms of the human microbiota, the majority of the research has been performed on the gut microbiome, which influences critical processes, such as digestion and the biosynthesis of vitamins [15]. To date, the gut microbiome is the most thoroughly researched one of all the body’s systems.

Changes at the gut microbiome level may be present in Pca patients, as shown by a pilot study conducted by Golombos and colleagues. The researchers discovered that those suffering from Pca had a significantly greater prevalence of *Bacteroides massiliensis* compared to individuals with benign prostatic hypertrophy. On the other hand, benign controls had significantly higher concentrations of *Eubacterium rectale* and *Faecalibacterium prausnitzii* compared to Pca patients. This discovery led to debates regarding the role of gut bacteria in this population [17].

Interestingly, in the research, Michael A. Liss et al. collected rectal swabs from 105 patients. Of these, 64 were confirmed with Pca, while 41 were non-cancerous patients. First, they measured the alfa diversity between these groups and found no association between them; however, when they focused on the beta diversity, the results showed an altered gut microbiota composition linked to Pca. They also analyzed *Bifidobacterium* (usually associated with healthy flora) and did not observe a significant difference between cancer patients and controls. At the species level, significant differences were observed between groups when *Bacteroides* and *Streptococcus* species were investigated. Using PICRUSt bioinformatics software, the authors observed that B vitamins (folate, biotin, and riboflavin) were significantly lacking in Pca patients; however, starch and sucrose metabolism were highly expressed in the same group. They identified the microbiome score consisting of 10 microbiome metabolic pathways, which resulted in an AUC = 0.64 compared to PSA = 0.5. The authors concluded that prostate-biopsied patients with cancer and those without cancer had similar fecal microbiomes. Although the metabolic pathways of the microbiome produced intriguing biological insights, they are unlikely to develop a “cancer” microbiome prediction risk profile as a result of these pathways. The development of a new 10-microbiome metabolic pathway scoring system could provide another potentially customizable Pca risk factor. Within the groups of bacteria, subtle changes in specific metabolic pathways were observed, especially for B vitamins, which may be useful for diagnosing high-risk individuals [18].

Multiple data exist to suggest that Pca risk may be influenced by dietary regimens. Nutrition itself has the greatest impact on the gut microbiota, where bad dietary routines can induce changes linked to increased inflammatory and tumorigenic activities. Inversely, favorable lifestyle modifications have been shown to lower the risk of various illnesses [19]. For example, a study conducted on animal subjects showed that a high-fat diet and obesity enhanced local prostate inflammation, Pca proliferation, and that short-chain fatty acids, important metabolites of gut bacteria, stimulated Pca development via the IGF1 signaling pathway [20].

The gut microbiota is responsible for many circulating vitamin syntheses involving bacterial metabolism, and these have a significant role in multiple physiological processes [21]. The bacterial production of vitamins can affect both host metabolism [22] and micro-organism communities [23]. There is ongoing research investigating the relationship between circulating vitamins involved in bacterial metabolism and prostate cancer. Vitamins, such as biotin, cobalamin, folates, nicotinic acid, pantothenic acid, pyridoxine, riboflavin, and thiamine, which are crucial for bacterial metabolism, can be produced by gut bacteria [21,24]. While these vitamins are primarily essential for bacterial growth and metabolism, there is evidence suggesting that certain metabolic and physiological processes in humans are also affected by their presence. For example, arginine, folate (vitamin B9), biotin (vitamin B7), and riboflavin (vitamin B2) are all produced by bacteria in the colon, and research has been conducted on the relationship between these nutrients and the risk of Pca in recent years [25,26,27]. It is important to mention that mammals do not synthesize B vitamins, and these are provided by diet or microbial sources. Since some bacterial species cannot synthesize these molecules, but need them for growth and survival, there are two ways to provide them with B vitamins. The direct way is through the intake of B vitamins via the diet, while the indirect process occurs when other species can support B-vitamin synthesis [22].

As a specific example, folate is a limiting factor in DNA methylation cycle, repair, and synthesis processes, and abnormal levels may impact cancer development via altered methylation patterns. Its intestinal production is crucial to maintaining metabolic homeostasis [28].

An extended study was conducted on a total of 6875 Pca patients and 8104 controls who were part of ATBC (the Alpha-Tocopherol, Beta-Carotene Cancer Prevention Study), CARET (Carotene and Retinol Efficacy Trial), EPIC (European Prospective Investigation into Cancer and Nutrition), NSHDC (Northern Sweden Health and Disease Study cohort), and ProtecT (Prostate Testing for Cancer and Treatment Trial), which investigated the relationship between folate levels and Pca development. They concluded that circulating folate concentration and Pca risk was grade-dependent, with an increased folate concentration being associated with a greater risk of high-grade illness. This association was not conclusive for low-grade diseases [29].

The microbiome’s capacity to influence systemic hormone levels may be crucial to treating the illness because estrogen and androgen levels affect Pca in a dual way [30]. There is mounting evidence that dietary components that positively alter the structure of the gut microbiota have a substantial effect on androgen exposure to the prostate and contribute to the decrease in Pca risk and development [31]. Furthermore, studies have shown a link between the gut microbiome and estrogen-driven malignancies, such as Pca, including estrogen-driven breast cancer, according to a group of gut bacteria that can break down estrogens (known as the estrobolome). It is known that Pca is hormone-sensitive and estrogens are critical to defining the anti-androgenic impact. Estrogens block the hypothalamus luteinizing hormone (LHRH), causing the pituitary gland to produce the luteinizing hormone (LH) [32,33]. It has been hypothesized that estrogen can interfere with cell division. The estrobolome has garnered a lot of attention, to date, based on these principles [34,35]. According to the research, Pca cell division is inhibited by exogenous estrogens, such as unmetabolized diethylstilbestrol (DES) [30].

Hormones and microbiota may operate independently in the development of illnesses and diseases; however, the research, at present, suggests that hormones and microbiota may work together in circumstances involving hormone hypersecretion or shortage [36]. As an example, Ridlon et al. demonstrated that *Clostridium scindens* can convert main bile acids into harmful secondary acids and remove the two-carbon side chain of glucocorticoids, converting them into androgen [37]. A significant relationship between microbiota and hormones was observed by Shin et al. in a cohort of males where testosterone levels were related to elevated levels of *Acinetobacter*, *Dorea*, *Ruminococcus*, and *Megamonas* [38]. All things considered, the possible roles of the microbiome in influencing hormone levels require further investigation.

The gut microbiome plays an important role in anti-cancer drug effectiveness and immunological modulation. Immune checkpoint blockades, such as PD1, PD-L1, and CTLA-4, are effective against numerous malignancies, including melanomas. The effectiveness and toxicity of immune checkpoint blockade cancer treatments are affected by various factors, including gut flora. Different gut microbiota compositions regulate anti-CTLA-4 or anti-PD-1/PD-L1 immunotherapy effectiveness. In this scenario, fecal microbiome transplantation (FMT) may impact the effectiveness of immune checkpoint blockades and be employed in immunotherapy in most circumstances. It is important to consider that gut microbiota and immune checkpoint inhibitor responsiveness are cohort-dependent [39].

FMT is a procedure that aims to control cancer patients’ microbiota and, interestingly, it permits the transfer of an entire patient’s gut microbiota to another [40,41]. The utility of FMT in modulating immunotherapeutic response has recently been investigated concerning multiple malignancies. For example, FMT for patients responding to immune checkpoint inhibitors ameliorated the antitumor efficiency of the PD-1 blockade and was correlated with increased IL-12 and the subsequent recruitment of CCR9^+^CXCR3^+^CD4^+^ T lymphocytes in in vivo models [42].

Early successes in acute myeloid leukemia and metastatic melanomas have prompted researchers to test this method on Pca patients who developed metastatic diseases following previous chemotherapy and radiation treatments. A phase-II single-arm experiment is presently testing the effectiveness of an FMT in metastatic castration-resistant Pca that has become resistant to the PD-1 inhibitors pembrolizumab and enzalutamide (NCT04116775). Pca patients were treated with enzalutamide administered given the immune checkpoint inhibitor pembrolizumab (200 mg flat dosage) for four cycles throughout the experiment, according to the study’s design. Responders then donated their feces to non-responders, who were subjected to a second biopsy and a re-challenge with pembrolizumab. A total of 32 people were enrolled in the research, where the main completion date was set for October 2022. In addition, the standard of care for Pca patients was enzalutamide and abiraterone acetate, the former of which binds directly to the androgen receptor and the latter of which targets testosterone production. Both medications mentioned above are taken orally and poorly soluble; therefore, they spend a long period of time in the presence of gut bacteria. As a result, the likelihood of microbial alteration is increased. Abiraterone acetate’s role may serve as a carbon source for bacteria [43].

Microbiota can affect drug metabolism in two ways: by either increasing or decreasing the activity of the drug. For example, in patients with prostate cancer, the microbiome may produce compounds that reduce the effectiveness of the drug. This can be detrimental for patients who have low natural testosterone levels. Previous research has shown that a human gut bacterium species called *Clostridium scindens* can convert glucocorticoids into androgens. This suggests that androgens can originate from both the host’s endocrine system and gut flora.

Moreover, disturbances in the gut microbiota produced by chemo- and immunotherapy have been proven to have an impact on the intestinal inflammatory milieu, systemic consequences of inflammation, and/or cancer therapeutic effectiveness in animal models [44]. Prokaryotes are responsible for steroid biosynthesis and certain bacterial species may break down estrogen and androgen precursors and catabolize them, affecting the systemic levels of these hormones [37,45].

Pca-bearing mice were studied by Pin-Yu Huang and colleagues, and fecal samples were collected from NOD/SCID (non-obese diabetic severe combined immunodeficient) mice to better understand the gut microbiota changes. Fecal samples were obtained from healthy controls and patients, starting four weeks after the seeding of Pca cells and weekly up to eight weeks. The first samples were obtained at 4 weeks, based on the hypothesis that tumor cells may be identified by IVIS at that time. QIIME2 was used to analyze 16S rRNA amplicon sequencing data to analyze microbiota profiles. All 30 samples had at least 976 amplicon sequence variations (ASVs) and 235 taxa. The alpha diversity between the 2 groups did not show significant differences compared to the beta diversity, which was significantly different between the groups, indicating that the microbiota composition between the 2 groups was significantly different. Another conclusion formed by the researchers was that, after the cancer cells’ inoculation, the microbiota started to diverge and it became significantly different as early as 4 weeks, with significant *p*-values observed for 14 of the 20 comparisons, at all-time points comparisons. *Akkermansiaceae* bacteria (inversely correlated with obesity, diabetes, inflammation, and metabolic disorders) was widely present in the tumor-bearing group compared to the controls; however, gradually, in 8 weeks, it became indistinguishable from the control group; there were also other bacterial abundances that correlated with the 2 groups [46].

In a research study, Sfanos et al. used 16S rDNA amplicon sequencing to characterize the fecal microbiota of 30 patients, including healthy male volunteers and men with localized metastatic Pca [47]. The results show that oral androgen receptor axis-targeted medications, such as bicalutamide, enzalutamide, and abiraterone acetate, promote an increase in the incidences of *Akkermansia muciniphila* and *Ruminococcaceae* spp. In the gut microbiota of men receiving these treatments. These two species, according to Gopalakrishnan V et al., are also related to anti-PD-1 treatment responses [48]. At present, immune checkpoint inhibitors are being intensively studied in patients with Pca; therefore, this topic is relevant in the context of understanding the potential of immunotherapy in Pca patients.

Radiotherapy is a treatment option for Pca patients that is widely used. Radiation-induced complications could be represented by radiation enteropathy, an event that may restrict the quantity of the dosed radiation. In an extended study, Ferreira et al. [49] included three cohorts of patients: the first one consisted of patients with early enteropathy (included before undergoing high-dose intensity-modulated radiotherapy to the prostate and pelvic lymph nodes (PLN-IMRT) and assessed at 2/3 weeks, and at 1, 3, 6, and 12 months); the second one included patients with late enteropathy (>2 years after irradiation for prostate and pelvic lymph nodes); and the third cohort consisted of patients with >1 year of enteropathy and controls (patients without enteropathy and Pca that only followed colonoscopy for colon cancer screening). Notably, radiation enteropathy patients had reduced bacterial diversity compared to patients without symptoms, patients before radiotherapy, and the controls. Additionally, radiation enteropathy patients had a higher prevalence of *Clostridium*, *Roseburia* and *Phascolarctobaterium* spp. (which are short-chain fatty acid producers, and their levels increased with symptoms of radiation enteropathy while interleukin 15 decreased). Therefore, further investigations of the relevance of altered microbiota in the development of radiation enteropathy that could possibly reduce such side effects are needed [50].

Chemotherapy can damage beneficial bacteria, such as *Bifidobacteria* and *Lactobacilli*, and, at the same time, permit the growth of potentially harmful ones (*Clostridia and Enterobacteriaceae*). Prebiotics can be administered as an attempt to alleviate gut imbalances by promoting the proliferation of probiotics, such as *Bifidobacteria* and *Lactobacilli*. It also increases the abundance and activity of specific gut microbial organisms present in the body and finally improves the bowel and immune systems [51]. At present, they are being studied as a therapeutic supplement to help mitigate intestinal dysbiosis caused by chemotherapy [52]. As far as gut microbiota restoration and immunotherapy responses are concerned, prebiotics might have a favorable impact on the therapeutic outcomes and quality of life by maintaining beneficial probiotics [51].

Table 1 presents a selection of the major microorganisms specific to the gut microbiota that have a potential influence on Pca development.

## 4. Urinary Microbiome and Pca

The urinary system was formerly assumed to be sterile and recent investigations have shown that the microbiome of the urine differs from that of the other sites of the body [53], being less abundant regarding diversity and the number of microorganisms [54]. Given the fact that the human body has various ways to defend itself against the bacterial infection of the urinary tract, most of the bacteria are washed away during urination if the urinary tract is unobstructed [55]. Moreover, urinary tract mucosa promotes this action by the secretion of organic acids, G and A immunoglobulins, and phagocytes [56], while the low pH of urine and its low or high tension is incompatible with bacterial growth [57]. In addition, prostatic fluid has similar bactericidal roles due to the high concentration of zinc ions [58].

Due to the anatomical proximity of the urinary system and prostate, the urine excretion pathway is interconnected within the prostate by the urethra where various microorganisms can grow and colonize this area, which can lead to the cancerous evolution of the surrounding cells. Microbial strains, such as *Corynebacterium*, *Streptococcus*, *Propionebacterium*, *Prevotella*, *Anaerococcus*, *Finegoldia*, *Staphylococcus*, and *Veillonella* genera, have been found in the urine of adult men in several investigations [59,60]. *Propionibacterium acnes*, recognized as a proinflammatory bacterium, is one of the most typically isolated bacteria from male urine and is associated with prostatitis, chronic inflammation, and Pca [7,60,61,62,63].

Moreover, the urinary tract microbial landscape has been shown to have a significant impact on different disease developments, such as urinary tract infections, urinary incontinence, chronic prostatitis, and chronic pelvic pain syndrome [64]. In this regard, the most abundant uropathogenic strains are represented by *Escherichia coli* and enterococci [65], especially from the *Clostridia* class [16,64]. Other commonly encountered uropathogens are *Corynebacterium glucuronolyticum*, *Streptococcus gallolyticus*, and *Aerococcus sanguinicola* [66,67,68]. Not only were bacteria observed to be responsible for urinary tract infections, but also various fungal species, such as *Clavispora lusitaniae*, *Candida albicans*, *C. orthopsilosis*, *C. tropicalis*, *C. glabrata*, *C. lusitaniae*, *Lodderomyces elongisporus*, *Meyerozyma guilliermondii*, and *Malassezia globose* [69,70].

Furthermore, urinary tract infection treatment responsiveness is affected by changes in the urinary microbiota [71]. Tolani et al. investigated the prevalence, risk factors, and antimicrobial sensitivity of pathogenic acute urinary tract infections in patients with benign prostate hyperplasia and Pca in 2016 and 2019. The most commonly encountered Gram-negative bacteria were *Escherichia coli*, *Klebsiella* spp., *Citrobacter* spp., and *Aerobacter* spp. From these, *E. coli* represented the principal cause of urinary tract infections with a high degree of sensitivity to antimicrobials, such as Nitrofurantoin [72]. The prevalence of such infections may be linked to urinary tract structural and functional abnormalities, which can promote *E. coli* infection due to low p-fimbrial adhesiveness [73]. Structural and functional urinary tract abnormalities promote chronic infections that lead to chronic inflammation. The latter is well documented as a malignant promoter.

In addition, Fan et al.’s research focused on the association between lower urinary tract infections and Pca development risks. Since these urinary tract infection symptoms overlap with Pca ones, the authors of these studies highly recommend evaluating the possibility of cystitis or urethritis as the first step in Pca diagnosis [74]. In these infectious conditions, the asymptomatic inflammation of the prostate can lead to Pca. A similar inflammatory mechanism is hypothesized to be related to sexually transmitted illnesses, especially in the cases of *Neisseria gonorrhoeae*- and *Chlamydia trachomatis*-positive patients [75,76,77]. As a consequence of the infections, these diseases are prone to affect the prostate’s epithelium, leading to inflammation and then increased PSA values, which, finally, may indicate prostate involvement [78].

A recent study conducted by Hurst et al. provides novel insights into the urine microbiome by describing the discovery of four new bacteria with potential prognostic values for Pca: *Porphyromonas* sp., *Varibaculum* sp., *Peptoniphilus* sp., and *Fenollaria* sp. [79]. These species were predominantly observed in the urine and prostate secretion samples of 300 patients following microbiome profiling involving metagenomics, DNA, and RNA sequencing. Another up-to-date study performed on urine samples isolated at different stages (before a biopsy and after a prostatectomy) focused on the development of a new strategy for microbial diagnostic biomarkers in Pca. Liss et al. used shotgun metagenomic sequencing technology, proteomics, and a paired comparison analysis, and identified eight microbial taxonomic signatures associated with PCa [80].

On the other hand, some of the microorganisms found in the urine can play an anti-tumoral role in PCa development. Some examples include *Bradyrhizobium japonicum*, *Listeria monocytogenes*, *Methylobacterium radiotolerans*, *Pseudomonas aeruginosa*, *Stenotrophomonas maltophilia*, and *Xanthomonas albilineans* [81]. They were negatively correlated with the Gleason score, Tumor, Node, Metastasis PSA (TNM PSA) levels, and androgen expression receptors. Of the previously listed organisms, *Listeria monocytogenes* has been involved in cancer immunotherapy research; it can induce apoptotic cell death through the increased production of intracellular reactive oxygen species [81].

*Propionibacterium* spp. and *Staphylococcus* spp. were more present in tumors and peri-tumoral features, compared to normal prostatic tissue, and this difference was clinically significant (*p* < 0.05). It was suspected that, when the above-mentioned bacteria outcompeted the natural microbiome of the prostate, chronic inflammation and immune imbalances could appear, damaging the DNA and precursing a PCa. Another pathway suggested for tumoral transformation and progression was the suppression of immune cell expression, which can be caused by bacteria, such as *E. coli*, *Campylobacter concisus*, *Streptococcus pneumoniae*, *Nevskia ramosa*, *Staphylococcus aureus*, *Paraburkaholderia phymatum*, *Gardnerella vaginalis*, and *Nitrobacter hamburgensis* [81].

One study characterized the urinary bacterial composition of 129 men, 63 of them with benign prostate biopsies and 61 with biopsy-confirmed PCa. They were compared the urine samples and observed no differences in terms of alpha and beta diversity values. In the cancer group, a cluster of pro-inflammatory species, such as *Streptococcus anginosus*, *Anaerococcus lactolyticus*, *Anaerococcus obesiensis*, *Actinobaculum schaalii*, *Varibaculum cambriense*, and *Propionimicrobium lymphophilum*, were highly abundant, compared to the control group [82].

When comparing PCa patients with healthy patients, the first group was found to have increased *Veillonella*, *Streptococcus*, and *Bacteroides* bacteria and decreased *Faecalibacterium*, *Lactobacilli*, and *Actinetobacter* bacteria compared to the controls. Additionally, *Clostridium XVIII* and *IV*, *Lachnospira*, *Acetanaerobacterium*, and *Faecalibacterium* were present in higher concentrations in the controls [83]. *E. coli* seemed to be reduced in PCa patients compared to the controls, and the results was the same for *Enterococcus* [84].

A summary of the microorganisms that colonize the urinary system and are related to PCa occurrence and progression is presented in Table 2.

However, further studies are still needed to learn about the involvement of urine microbiota in the development and progression of PCa; therefore, they are eagerly anticipated. The possibility of contamination and the standardization of methods and processes for sample collection and analysis poses significant challenges in this respect [85]. In this regard, many studies have focused on profiling urinary microbiome for discriminating between the positive and negative diagnosis of PCa using high-throughput technologies, such as the DNA sequencing of bacterial 16S rRNA genes [71,82].

## 5. Prostate Microbiome and PCa

Approximately 80% to 90% of adult men have some type of inflammation at the prostate level at some point in their lives. In these cases, there is an increase in the number of prostatic immune cells. Increased inflammatory cell infiltration into the prostate is frequent, although it is thought to be a risk factor and contributes to the majority of cases of prostate disease [14].

The function of the microbiota in enhancing the status of chronic inflammation and its potential involvement in the development of PCa is becoming clearer [16]. This theory is supported by the finding of a urinary microbiome that contains a diverse range of microorganisms, since the prostate is exposed to many inflammatory stimuli generated by the bacteria in this environment. Since the prostate is located next to the urethra, it is exposed to bacteria from the urinary system [82]. In the pathological context of the prostate, due to the presence of pathogenic bacteria, the epithelial barrier is breached, allowing for the proliferation of bacteria and inflammation, which results in an immune response that causes DNA damage, cell injury, and cell death. This results in a persistent inflammatory state that encourages epithelial cell regeneration, producing proliferative inflammatory atrophy (PIA), which transitions into low- and high-grade prostate intraepithelial neoplasia (PIN), and then into prostate adenocarcinoma [14].

Recent research has evaluated the roles of urogenital microbiota in several urogenital disorders, both benign and malignant, including urinary tract infections, chronic prostatitis, urinary incontinence, interstitial cystitis, and bladder, prostate, and kidney cancers [86]. Intraprostatic inflammation originates from many causes, including infection, injury, hormone fluctuation, exposure to chemicals, and physical stress. A potential explanation for an autoimmune response may be the presence of epithelial cellular damage, which can lead to tolerance loss to typical prostatic antigens [14].

Normal prostate cells, as well as cancer cells, need androgens for survival and growth. In this regard, androgen deprivation therapy is a common therapeutic option for this type of cancer. Compared to normal prostatic tissues, PCa cells develop independence from androgen deprivation, and, at some point, they restart growth and metastasis. The effectiveness of the therapy mentioned above can be influenced by the microbiome because of its capacity to metabolize estrogens and androgens [10].

Regarding the study mentioned above, the researchers discovered a unique microbial landscape in individuals who received oral androgen suppression medication compared to those who did not. An increment in the bacterial metabolic pathways that drive androgen production was observed in treated individuals. According to the findings, substantial changes in the gut microbial structure were discovered when comparing PCa patients and individuals with benign tumors [47].

More recent research has investigated the function of bacteria as infectious agents instead of natural flora in PCa. For instance, a possible cause of PCa has been identified for sexually transmitted diseases (STDs) related to bacterial species, such as *Chlamydia trachomatis* [87]. It has been hypothesized that males with a promiscuous sexual activity and multiple episodes of SDTs could be at a higher risk of developing PCa; however, a high ejaculatory frequency appears to play a protective role against PCa. *Trichomonas vaginalis* and *Chlamydia trachomatis* could create a microenvironment favorable for PCa development [81].

Polymerase chain reaction (PCR) and 16S rRNA sequencing have been used to identify host factors, such as the upregulation of receptors and mother-to-child bacterial transmission in the first few months of life, which may contribute to the colonization and survival of these bacteria in the urinary tract without evidence of infection [88].

Miyake et al. conducted a study in which 45 PCa samples and 33 benign prostatic hyperplasia (BPH) samples were tested for different sexually transmitted infectious agents using PCR. Only *Mycoplasma genitalium* was shown to be independently related to PCa and with high Gleason scores. This study indicated that carcinogenic microorganisms were present in prostate tumor tissue, which may be related to prostatic inflammation and tumorigenesis [89].

Additionally, it was shown that the DNA profiles of viruses, bacteria, and fungi were all present in the formalin-fixed tissue of 50 men with PCa and 15 men with BPH in a study by Banerjee et al. in 2019. In decreasing order of frequency, the most prevalent bacterial signatures were *Proteobacteria*, *Firmicutes*, *Actinobacteria*, and *Bacteroidetes*. One-fourth of the viruses found were recognized as tumor-causing ones, such as human papillomavirus types 16 and 18 (HPV-16&18) and human cytomegalovirus type 1 (HCMV-1). Gleason scores were shown to be lower among those who tested positive for HPV-18, Kaposi’s sarcoma-associated herpes virus (KSHV), or *Polyomaviridae* [90].

The stimulator of interferon genes (STING) pathway is known to promote viral immunological tolerance. Few data address the function of this pathway in PCa etiology. However, polyomaviruses, human papillomavirus (HPV), and human cytomegalovirus (HCMV) infections have all been linked to PCa [91,92]. Cytomegalovirus (CMV) was discovered in individuals with PIN. Following the BK virus (BKV), polyomaviruses (John Cunningham virus—JCV), HPVs, and Epstein–Barr viruses (EBVs) were shown to be connected to prostatic cancers, continuing with the other research that supports these findings [93]. For example, Banerjee S. et al. discovered HPV-18, CMV, KSH, EBV, BKV, and JCV in PCa samples. BPH specimens also included HPV-18 and KSH viruses [90]. Significantly, HPV-18 and the KSH virus were found to be incorporated into the host genome most often. HPV-18 has a documented involvement in the development of squamous cell carcinomas and adenocarcinomas in the cervix, which adds an interesting level of complexity to the potential link between it and PCa. The fact remains, however, that these viruses were discovered in BPH samples, however, it was difficult to determine the causative role since the time of processing it was unclear. They also employed next-generation sequencing to examine the microbiome inside prostate tissue samples from PCa and BPH and discovered three distinct microbial signatures that were associated with the PCa stage, grade, and Gleason score. Additionally, they discovered that the PCa microbiome was diverse in terms of viruses, fungi, bacteria, and parasites that were differently recognized, and they all presented specific signatures [90]. This contrasts with the findings of Feng and colleagues, who discovered no significant variations in the number of bacterial taxa in prostate tissue samples from individuals with and without PCa [94]. Clearly, there are considerable microbiome variations across patients, making the confirmation of any microbial fingerprints problematic.

Approaching the immune system, the tumor microenvironment increases the infiltration of several immune cell types, such as neutrophils, macrophages, dendritic cells (DCs), adipose cells, T cells, B cells, and natural killer cells (NKs). Similar to many others, immune systems have been found to be controlled by microbes. The inflammatory response associated with chronic infections consists of a reaction cascade that determines the release of chemotactic cytokines. In turn, this results in the local accumulation of immune cells, such as neutrophils, which release antimicrobial agents, such as reactive oxygen species (ROS), neutrophil extracellular traps, antibacterial peptides, and enzymes. Continuous ROS accumulation due to chronic inflammation can affect the local environment, given its susceptibility to ROS-determined DNA damage, indirectly promoting oncogenesis [95].

An important component of the TME and one of the most abundant cells type in virtually all solid tumors, cancer-related fibroblasts (CAFs) promote tumor development, especially via the TGF-beta pathway. The crosstalk between CAFs and tumor cells can occur either via direct contact or secreted factors and can functionally inhibit immunosurveillance by suppressing CD8+ T-cell- and NK-cell-mediated anti-tumor immune responses [96]. At present, there are indications that the enrichment of specific microorganisms in the prostate microbiome can influence immune and stromal architecture. For example, by using whole-transcriptome profiling, Salachan et al. indicated that differential counts of *Shewanella* and *Vibrio parahaemolyticus* had lower enrichment scores for fibroblasts and significantly higher ones for monocytes, B cells, CD8+ T cells, mast cells, M2 macrophages, and T-regulatory cells, respectively. *Shewanella* genera was present in malignant prostate tissue samples compared to benign prostate tissue samples [97]. Moreover, it was possible that other prostate-specific bacteria mediated tumor sensitivity to immunotherapy. It is known that *Trichomonas vaginalis* can increase the risk for development of PCa by activating cytokines IL-6, IL-8, and NF-κB. Specifically, epithelial prostate cells stimulated by *Trichomonas vaginalis* can determine the M2 polarization of macrophages via secreted IL-6, which in turn activates the secretion of IL-10 and TGB-Beta [98]. These cytokines engage with the macrophage migration inhibitory factor, Proto-oncogene (PIM 1), and PSA to polarize M2 macrophages and induce the proliferation of PCa [98,99].

Another bacteria, *Gardnerella Vaginalis*, also sexually transmitted, can determine the downregulation of immune-regulating genes LPCAT2, TLR3, and TGF B2 genes, which can enhance the development of PCa by modulating the macrophage inflammatory gene expression [100].

Similarly, the Influenza A virus, *Staphylococcus aureus*, and *Streptococcus* group A can activate the expression of the transforming growth factor (TGF) to promote tumor cell proliferation and metastasis [101,102].

Table 3 presents a short description of the microorganisms presented, their origin, roles, and an abundance comparison between normal tissue vs. PCa samples.

## 6. Oral and Skin Microbiomes in Relation to PCa

Several recent studies have shown that poor oral hygiene results in the destruction of oral bacterial populations, which increases the frequency of oral pathogens. Previously, it was shown that oral infections are associated with a variety of disorders, including cardiovascular disease [104], and pancreatic cancer [105], among other pathologies. The connection between the oral microbiome and the risk of developing PCa is still poorly described; however, there is a theory that pathogenic bacteria may migrate from the mouth to other regions of the body in a range of disorders, including prostatitis. In addition, multiple investigations have shown a link between periodontitis and elevated prostate-specific antigen (PSA) levels [106]. Additionally, there is a link between PSA levels and chronic prostatitis, and periodontitis cohabitation. Increased blood PSA levels may also relate to PCa’s aggressiveness [107]. Thus, by taking into consideration the fact that PCa and periodontitis have similar etiopathogenesis effects, first oral infectious disorders may induce widespread inflammation throughout the body by elevating C-reactive protein (CRP) levels as well as proinflammatory cytokines, such as tumor necrosis factor (TNF), IL-1, and IL-6 levels, which may, in turn, result in prostate inflammation [108].

A meta-analysis by Corbella et al. identified a statistically significant link between smoking and all the malignancies evaluated, both together and separately, for the digestive tract, pancreas, prostate, and non-Hodgkin’s lymphoma [109].

There is a link between prostatitis and periodontitis, and the presence of oral bacterial DNA in the prostate secretions of males with both disorders suggests that inflammatory processes may be involved in both chronic conditions [11]. As a result, eliminating the oral foci of infection is a critical objective in the treatment of PCa [110]. *Porphyromonas gingivalis* was observed to be the most frequently isolated pathogen obtained from prostatic secretions. This bacterium has the capacity to create Arg-gingipain, which is a collagen-degrading substance that can penetrate epithelial cells via binding to erythrocytes, which allows it to spread throughout the body [103]. As a result of its ability to disrupt signaling pathways, *P. gingivalis* has the potential to infiltrate the human immune system. Patients with a clinical attachment level (gingival retraction) ≥ 2.7 mm and concomitant moderate/severe prostatitis had higher PSA levels than individuals who just had one of these diseases, according to previous research by Joshi et al. [107].

Additionally, a recent study that included individuals with chronic prostatitis and periodontal illnesses observed a strong and positive link between PSA levels, as well as clinical attachment level scores, probing depth, gingival index, and plaque index values [111]. Patients with periodontal disease had lower PSA levels than males without PCa, inflammation, or infection, even after considering their age and other risk factors. However, the multivariable-adjusted odds ratios demonstrated that the difference between patients with severe periodontal disease and healthy controls was negligible when it came to having PSA levels higher than 4.0 ng/mL [112]. According to Alwithanani et al. [110], periodontal therapy resulted in lowering the PSA levels in males with aberrant prostate tissue observed during a digital rectal exam. Following periodontal therapy, there was a statistical link between changing PSA levels and periodontal markers, such as clinical attachment level, gingival inflammation, BOP, bleeding on probing, and gingival recession.

Conflicting information was presented in a prospective study conducted on 47 patients with chronic periodontitis without a previous diagnosis of prostate pathologies or therapy (asymptomatic and PSA values of 4 ng/mL), determining that the periodontal treatment had no effect on PSA levels [113]. Together, these data show that PSA levels in men with symptomatic prostate illness may be linked to the severity of their periodontal disease; however, further clinical research is needed.

Regarding the skin microbiome, it was shown that *Proprionibacterium acnes*, an anaerobic Gram-positive bacteria present in human sebaceous follicles, causes inflammatory disorders via hemolytic, cytotoxic, and immunomodulatory actions. This bacterium was observed in high concentrations in the samples obtained from patients with metastatic PCa [114,115]. According to the research, PCa-related *P. acnes* strains differed in surface features from *P. acnes* strains linked with skin pathologies and have been reported to have potent inflammatory properties in prostate tissue. The activation of nuclear factor Kappa B (NF-κB), IL-6-Stat3, and COX2-PGE2 pathways, as well as the enhanced transcriptional activation of the IL-8 and VEGF genes were observed in experiments performed in vitro on RWPE-1 cell lines infected with *P. acnes* [116]. This bacterial infection caused the main NF-κB inhibitor alpha (IκBα) breakdown and NF-κB activation, positively regulating the genes involved in PCa growth and progression processes [116]. Furthermore, elevated IL-6 levels in the PCa serum were linked to progressive metastases and stimulated JAK/STAT signaling, which may lead to tumor growth and proliferation [117].

## 7. Conclusions

It seems that the microbiome may have a significant impact on the diagnosis, development, and treatment of PCa, and additional research is required to confirm this result. The pre-clinical and clinical evidence reveal that microbial organisms play a major role in the development and progression of numerous diseases, even though these interactions are not fully understood.

However, the research on prostate cancer microbiomes still faces several limitations, such as a lack of standardized sample-collection and analysis methods, conflicting results from different studies, and the requirement of larger, more diverse study populations to increase the generalizability of the results. Additionally, this is a controversial topic due to a lack of data that connects host microbial species to their functional profiles and describes the specific ways in which microbes either promote cancer development and progression or support antitumor immunity. While only a small number of microbes are known to directly cause cancer, several others have been observed to aid in host antitumor immunity.

There is a deficit of published studies on the urinary microbiome’s participation in other genitourinary malignancies, such as penile and testicular cancers, even though several studies have examined its role in cancers of the bladder, kidneys, and prostate. Anti- and probiotics, for example, may be used to influence the microbiome for therapeutic reasons in the future. To bring these concepts closer to regular clinical applications and explore how bacterial abundance affects disease processes in the microbiome and genitourinary cancer, well-designed prospective or systemic approaches are required. The interaction of viruses, fungi, and bacteria in promoting disease development and treatment responses is still poorly described; however, it might be a useful tool for identifying individuals with a high risk of disease progression or treatment failure.

The association between microbiomes from the gut–prostate axis is much more challenging to study due to the difficulties of sample bias, determining the species that interact with the prostate, and what factors can induce tumoral growth. Future research with better sampling techniques may be able to address these issues and provide relevant information, if microbiota-targeted therapies are necessary and can improve treatment responses.

To conclude, the microbiome may play a key role in the diagnosis, development, and therapy of PCa; however, additional research is required to clarify these processes.

## Figures and Tables

**Table 1:** Summary of relevant gut microorganisms related to Pca development.

Microorganisms	Isolation Site	Basic Involvement in Pca	Normal Tissue/BPH Samples	Pca Samples	Ref.
*Bacteroides massiliensis*	gut	processes complex molecules	low levels	high abundance	[17]
*Eubacterium rectale*	gut	major producer of butyrate	high concentration	low concentration	[17]
*Faecalibacterium prausnitzii*	gut	produces butyrate from its utilization of acetate anti-inflammatory properties	high concentration	low concentration	[17]
*Clostridium scindens*	gut	converts glucocorticoids into androgens	present	could interfere with androgen deprivation therapy	[37]
*Akkermansiaceae*, *Ruminococcaceae* spp.	Gut	response to anti-PD1 immunotherapy	present	widely present; high concentration in men with androgen deprivation therapy	[47]
*Clostridium*, *Roseburia*, *Phascolarctobacterium* spp.	Gut	short-chain fatty acid producers	present	high prevalence in radiation enteropathy patients	[50]
*Bifidobacteria* and *Lactobacilli*	gut	increase the abundance and activity of specific gut microbial organisms	present	decreased	[52]

**Table 2:** Urinary system microorganisms linked to PCa development.

Microorganisms	Isolation Site	Basic Involvement in PCa	Normal Tissue/BPH Samples	PCa Samples	Ref.
*Proprionibacterium acnes*	urine	proinflammatory	present	increased in prostatitis, chronic inflammation, and PCa	[60]
*Porphyromonas* sp., *Varibaculum* sp., *Peptoniphilus* sp., and *Fenollaria* sp.	urine and prostate secretion	prognostic value for PCa	present	high in patients with high-risk PCa	[80]
*Bradyrhizobium japonicum*, *Listeria monocytogenes*, *Methylobacterium radiotolerans*, *Pseudomonas aeruginosa*, *Stenotrophomonas maltophilia* and *Xanthomonas albilineans*	urine	anticancer properties	present	low	[81]
*Propionibacterium* spp. and *Staphylococcus* spp.	Urine	infectious	absent	present	[81]
*E. coli*, *Campylobacter concisus*, *Streptococcus pneumoniae*, *Nevskia ramosa*, *Staphylococcus aureus*, *Paraburkaholderia phymatum*, *Gardnerella vaginalis*, *Nitrobacter hamburgensis*	urine	chronic inflammation, immune imbalance, DNA damage	absent	present	[81]
*Neisseria gonorrhoeae* and *Chlamydia trachomatis*	urine	infectious	absent	present	[77]
*Streptococcus anginosus*, *Anaerococcus lactolyticus*, *Anaerococcus obesiensis*, *Actinobaculum schaalii*, *Varibaculum cambriense* and *Propionimicrobium lymphophilum*	urine	pro-inflammatory	absent	abundant	[82]
*Veillonella*, *Streptococcus*, *Bacteroides*	urine	part of the normal flora of the mouth and gastrointestinal tract	low	abundant	[83]
*Aecalibacterium*, *Lactobacilli*, *Actinetobacter*	urine	abundant and important commensal bacteria	present	decreased	[83]

**Table 3:** Prostate-colonizing microorganisms involved in PCa development.

Microorganisms	Isolation Site	Basic Involvement in PCa	Normal Tissue/BPH Samples	PCa Samples	Ref.
*Trichomonas vaginalis* and *Chlamydia trachomatis*	prostate	M2 macrophage polarization affects mRNA expression for IL-6 and FGF-2	absent	present	[81]
*Mycoplasma genitalium*	prostate	causes sexual transmitted diseases	absent	present	[89]
*Proteobacteria*, *Firmicutes*, *Actinobacteria*, and *Bacteroidetes*	prostate	infection	absent	abundant	[90]
Polyomaviruses, human papillomaviruses (HPVs), human cytomegalovirus	prostate	infection	absent	present	[91]
*Porphyromonas gingivalis*	prostatic secretion	secreting Arg-gingipain, a collagen-degrading substance	absent	present	[103]

## References

[1] Ferlay J., Colombet M., Soerjomataram I., Parkin D.M., Piñeros M., Znaor A., Bray F. (2021). Cancer statistics for the year 2020: An overview. Int. J. Cancer.

[2] Sung H., Ferlay J., Siegel R.L., Laversanne M., Soerjomataram I., Jemal A., Bray F. (2021). Global Cancer Statistics 2020: GLOBOCAN Estimates of Incidence and Mortality Worldwide for 36 Cancers in 185 Countries. CA. Cancer J. Clin..

[3] Drummond F.J., Carsin A.-E., Sharp L., Comber H. (2009). Factors prompting PSA-testing of asymptomatic men in a country with no guidelines: A national survey of general practitioners. BMC Fam. Pract..

[4] Franco G.S., Hardie R., Li L., Imai C., Sezgin G., Li J., McLeod A., Pearce C., Georgiou A. (2021). Prostate-specific antigen testing of asymptomatic men in Australia: An observational study based on electronic general practice data. Med. J. Aust..

[5] Biddle C., Brasel A., Underwood W., Orom H. (2017). Experiences of Uncertainty in Men With an Elevated PSA. Am. J. Mens. Health.

[6] Schatteman P.H.F., Hoekx L., Wyndaele J.J., Jeuris W., Van Marck E. (2000). Inflammation in Prostate Biopsies of Men without Prostatic Malignancy or Clinical Prostatitis. Eur. Urol..

[7] Shinohara D.B., Vaghasia A.M., Yu S.-H., Mak T.N., Brüggemann H., Nelson W.G., De Marzo A.M., Yegnasubramanian S., Sfanos K.S. (2013). A mouse model of chronic prostatic inflammation using a human prostate cancer-derived isolate of *Propionibacterium acnes*. Prostate.

[8] Alfano M., Canducci F., Nebuloni M., Clementi M., Montorsi F., Salonia A. (2016). The interplay of extracellular matrix and microbiome in urothelial bladder cancer. Nat. Rev. Urol..

[9] Javier-DesLoges J., McKay R.R., Swafford A.D., Sepich-Poore G.D., Knight R., Parsons J.K. (2022). The microbiome and prostate cancer. Prostate Cancer Prostatic Dis..

[10] Porter C.M., Shrestha E., Peiffer L.B., Sfanos K.S. (2018). The microbiome in prostate inflammation and prostate cancer. Prostate Cancer Prostatic Dis..

[11] Estemalik J., Demko C., Bissada N.F., Joshi N., Bodner D., Shankar E., Gupta S. (2017). Simultaneous Detection of Oral Pathogens in Subgingival Plaque and Prostatic Fluid of Men With Periodontal and Prostatic Diseases. J. Periodontol..

[12] Roy S., Trinchieri G. (2017). Microbiota: A key orchestrator of cancer therapy. Nat. Rev. Cancer.

[13] Colotta F., Allavena P., Sica A., Garlanda C., Mantovani A. (2009). Cancer-related inflammation, the seventh hallmark of cancer: Links to genetic instability. Carcinogenesis.

[14] De Marzo A.M., Platz E.A., Sutcliffe S., Xu J., Grönberg H., Drake C.G., Nakai Y., Isaacs W.B., Nelson W.G. (2007). Inflammation in prostate carcinogenesis. Nat. Rev. Cancer.

[15] Lynch S.V., Pedersen O. (2016). The Human Intestinal Microbiome in Health and Disease. N. Engl. J. Med..

[16] Sfanos K.S., Yegnasubramanian S., Nelson W.G., De Marzo A.M. (2018). The inflammatory microenvironment and microbiome in prostate cancer development. Nat. Rev. Urol..

[17] Golombos D.M., Ayangbesan A., O’Malley P., Lewicki P., Barlow L., Barbieri C.E., Chan C., DuLong C., Abu-Ali G., Huttenhower C. (2018). The Role of Gut Microbiome in the Pathogenesis of Prostate Cancer: A Prospective, Pilot Study. Urology.

[18] Liss M.A., White J.R., Goros M., Gelfond J., Leach R., Johnson-Pais T., Lai Z., Rourke E., Basler J., Ankerst D. (2018). Metabolic Biosynthesis Pathways Identified from Fecal Microbiome Associated with Prostate Cancer. Eur. Urol..

[19] Pham V.T., Dold S., Rehman A., Bird J.K., Steinert R.E. (2021). Vitamins, the gut microbiome and gastrointestinal health in humans. Nutr. Res..

[20] Matsushita M., Fujita K., Motooka D., Hatano K., Fukae S., Kawamura N., Tomiyama E., Hayashi Y., Banno E., Takao T. (2021). The gut microbiota associated with high-Gleason prostate cancer. Cancer Sci..

[21] Rowland I., Gibson G., Heinken A., Scott K., Swann J., Thiele I., Tuohy K. (2018). Gut microbiota functions: Metabolism of nutrients and other food components. Eur. J. Nutr..

[22] Yoshii K., Hosomi K., Sawane K., Kunisawa J. (2019). Metabolism of Dietary and Microbial Vitamin B Family in the Regulation of Host Immunity. Front. Nutr..

[23] Magnúsdóttir S., Ravcheev D., de Crécy-Lagard V., Thiele I. (2015). Systematic genome assessment of B-vitamin biosynthesis suggests co-operation among gut microbes. Front. Genet..

[24] Hill M.J. (1997). Intestinal flora and endogenous vitamin synthesis. Eur. J. Cancer Prev..

[25] Pieroth R., Paver S., Day S., Lammersfeld C. (2018). Folate and Its Impact on Cancer Risk. Curr. Nutr. Rep..

[26] Franko A., Shao Y., Heni M., Hennenlotter J., Hoene M., Hu C., Liu X., Zhao X., Wang Q., Birkenfeld A.L. (2020). Human Prostate Cancer is Characterized by an Increase in Urea Cycle Metabolites. Cancers.

[27] Bassett J.K., Severi G., Hodge A.M., Baglietto L., Hopper J.L., English D.R., Giles G.G. (2012). Dietary intake of B vitamins and methionine and prostate cancer incidence and mortality. Cancer Causes Control.

[28] Engevik M.A., Morra C.N., Röth D., Engevik K., Spinler J.K., Devaraj S., Crawford S.E., Estes M.K., Kalkum M., Versalovic J. (2019). Microbial Metabolic Capacity for Intestinal Folate Production and Modulation of Host Folate Receptors. Front. Microbiol..

[29] Price A.J., Travis R.C., Appleby P.N., Albanes D., Barricarte Gurrea A., Bjørge T., Bueno-de-Mesquita H.B., Chen C., Donovan J., Gislefoss R. (2016). Circulating Folate and Vitamin B12 and Risk of Prostate Cancer: A Collaborative Analysis of Individual Participant Data from Six Cohorts Including 6875 Cases and 8104 Controls. Eur. Urol..

[30] Cavalieri E., Chakravarti D., Guttenplan J., Hart E., Ingle J., Jankowiak R., Muti P., Rogan E., Russo J., Santen R. (2006). Catechol estrogen quinones as initiators of breast and other human cancers: Implications for biomarkers of susceptibility and cancer prevention. Biochim. Biophys. Acta-Rev. Cancer.

[31] Bland J.S. (2021). Prostate Cancer Risk Connection to Immunity, Hormones, and the Microbiome. Integr. Med..

[32] Nelles J.L., Hu W.-Y., Prins G.S. (2011). Estrogen action and prostate cancer. Expert Rev. Endocrinol. Metab..

[33] Wirén S., Stocks T., Rinaldi S., Hallmans G., Bergh A., Stenman U.-H., Kaaks R., Stattin P. (2007). Androgens and prostate cancer risk: A prospective study. Prostate.

[34] Komorowski A.S., Pezo R.C. (2020). Untapped “-omics”: The microbial metagenome, estrobolome, and their influence on the development of breast cancer and response to treatment. Breast Cancer Res. Treat..

[35] Ervin S.M., Li H., Lim L., Roberts L.R., Liang X., Mani S., Redinbo M.R. (2019). Gut microbial β-glucuronidases reactivate estrogens as components of the estrobolome that reactivate estrogens. J. Biol. Chem..

[36] Tetel M.J., de Vries G.J., Melcangi R.C., Panzica G., O’Mahony S.M. (2018). Steroids, stress and the gut microbiome-brain axis. J. Neuroendocrinol..

[37] Ridlon J.M., Ikegawa S., Alves J.M.P., Zhou B., Kobayashi A., Iida T., Mitamura K., Tanabe G., Serrano M., De Guzman A. (2013). Clostridium scindens: A human gut microbe with a high potential to convert glucocorticoids into androgens. J. Lipid Res..

[38] Shin J.-H., Park Y.-H., Sim M., Kim S.-A., Joung H., Shin D.-M. (2019). Serum level of sex steroid hormone is associated with diversity and profiles of human gut microbiome. Res. Microbiol..

[39] Xu H., Cao C., Ren Y., Weng S., Liu L., Guo C., Wang L., Han X., Ren J., Liu Z. (2022). Antitumor effects of fecal microbiota transplantation: Implications for microbiome modulation in cancer treatment. Front. Immunol..

[40] Chen D., Wu J., Jin D., Wang B., Cao H. (2019). Fecal microbiota transplantation in cancer management: Current status and perspectives. Int. J. Cancer.

[41] Wu X., Zhang T., Chen X., Ji G., Zhang F. (2019). Microbiota transplantation: Targeting cancer treatment. Cancer Lett..

[42] Routy B., Le Chatelier E., Derosa L., Duong C.P.M., Alou M.T., Daillère R., Fluckiger A., Messaoudene M., Rauber C., Roberti M.P. (2018). Gut microbiome influences efficacy of PD-1-based immunotherapy against epithelial tumors. Science.

[43] Maleki Vareki S., Chanyi R.M., Abdur-Rashid K., Brennan L., Burton J.P. (2018). Moving on from Metchnikoff: Thinking about microbiome therapeutics in cancer. Ecancermedicalscience.

[44] Fijlstra M., Ferdous M., Koning A.M., Rings E.H.H.M., Harmsen H.J.M., Tissing W.J.E. (2015). Substantial decreases in the number and diversity of microbiota during chemotherapy-induced gastrointestinal mucositis in a rat model. Support. Care Cancer.

[45] Bode H.B., Zeggel B., Silakowski B., Wenzel S.C., Reichenbach H., Müller R. (2003). Steroid biosynthesis in prokaryotes: Identification of myxobacterial steroids and cloning of the first bacterial 2,3(S)-oxidosqualene cyclase from the myxobacterium *Stigmatella aurantiaca*. Mol. Microbiol..

[46] Huang P.-Y., Yang Y.-C., Wang C.-I., Hsiao P.-W., Chiang H.-I., Chen T.-W. (2021). Increase in *Akkermansiaceae* in Gut Microbiota of Prostate Cancer-Bearing Mice. Int. J. Mol. Sci..

[47] Sfanos K.S., Markowski M.C., Peiffer L.B., Ernst S.E., White J.R., Pienta K.J., Antonarakis E.S., Ross A.E. (2018). Compositional differences in gastrointestinal microbiota in prostate cancer patients treated with androgen axis-targeted therapies. Prostate Cancer Prostatic Dis..

[48] Gopalakrishnan V., Spencer C.N., Nezi L., Reuben A., Andrews M.C., Karpinets T.V., Prieto P.A., Vicente D., Hoffman K., Wei S.C. (2018). Gut microbiome modulates response to anti–PD-1 immunotherapy in melanoma patients. Science.

[49] Reis Ferreira M., Andreyev H.J.N., Mohammed K., Truelove L., Gowan S.M., Li J., Gulliford S.L., Marchesi J.R., Dearnaley D.P. (2019). Microbiota- and Radiotherapy-Induced Gastrointestinal Side-Effects (MARS) Study: A Large Pilot Study of the Microbiome in Acute and Late-Radiation Enteropathy. Clin. Cancer Res..

[50] Nicolaro M., Portal D.E., Shinder B., Patel H.V., Singer E.A. (2020). The human microbiome and genitourinary malignancies. Ann. Transl. Med..

[51] Sambi M., Bagheri L., Szewczuk M.R. (2019). Current Challenges in Cancer Immunotherapy: Multimodal Approaches to Improve Efficacy and Patient Response Rates. J. Oncol..

[52] Wang H., Geier M.S., Howarth G.S. (2016). Prebiotics: A Potential Treatment Strategy for the Chemotherapy-damaged Gut?. Crit. Rev. Food Sci. Nutr..

[53] Whiteside S.A., Razvi H., Dave S., Reid G., Burton J.P. (2015). The microbiome of the urinary tract—A role beyond infection. Nat. Rev. Urol..

[54] Perez-Carrasco V., Soriano-Lerma A., Soriano M., Gutiérrez-Fernández J., Garcia-Salcedo J.A. (2021). Urinary Microbiome: Yin and Yang of the Urinary Tract. Front. Cell. Infect. Microbiol..

[55] Cox C.E., Hinman F. (1961). Experiments with Induced Bacteriuria, Vesical Emptying and Bacterial Growth on the Mechanism of Bladder Defense to Infection. J. Urol..

[56] Fowler J.E. (1991). Secretory immunity of the prostate gland. Infection.

[57] Ueda N., Kondo M., Takezawa K., Kiuchi H., Sekii Y., Inagaki Y., Soda T., Fukuhara S., Fujita K., Uemura M. (2020). Bladder urothelium converts bacterial lipopolysaccharide information into neural signaling via an ATP-mediated pathway to enhance the micturition reflex for rapid defense. Sci. Rep..

[58] Krieger J.N., Rein M.F. (1982). Zinc Sensitivity of *Trichomonas vaginalis*: In Vitro Studies and Clinical Implications. J. Infect. Dis..

[59] Nelson D.E., Van Der Pol B., Dong Q., Revanna K.V., Fan B., Easwaran S., Sodergren E., Weinstock G.M., Diao L., Fortenberry J.D. (2010). Characteristic Male Urine Microbiomes Associate with Asymptomatic Sexually Transmitted Infection. PLoS ONE.

[60] Dong Q., Nelson D.E., Toh E., Diao L., Gao X., Fortenberry J.D., Van Der Pol B. (2011). The Microbial Communities in Male First Catch Urine Are Highly Similar to Those in Paired Urethral Swab Specimens. PLoS ONE.

[61] Davidsson S., Mölling P., Rider J.R., Unemo M., Karlsson M.G., Carlsson J., Andersson S.-O., Elgh F., Söderquist B., Andrén O. (2016). Frequency and typing of *Propionibacterium acnes* in prostate tissue obtained from men with and without prostate cancer. Infect. Agent. Cancer.

[62] Simons B.W., Durham N.M., Bruno T.C., Grosso J.F., Schaeffer A.J., Ross A.E., Hurley P.J., Berman D.M., Drake C.G., Thumbikat P. (2015). A human prostatic bacterial isolate alters the prostatic microenvironment and accelerates prostate cancer progression. J. Pathol..

[63] Manente L., Gargiulo U., Gargiulo P., Dovinola G. (2022). *Propionibacterium acnes* in urine and semen samples from men with urinary infection. Arch. Ital. Urol. Androl..

[64] Shoskes D.A., Altemus J., Polackwich A.S., Tucky B., Wang H., Eng C. (2016). The Urinary Microbiome Differs Significantly Between Patients With Chronic Prostatitis/Chronic Pelvic Pain Syndrome and Controls as Well as Between Patients With Different Clinical Phenotypes. Urology.

[65] Khasriya R., Sathiananthamoorthy S., Ismail S., Kelsey M., Wilson M., Rohn J.L., Malone-Lee J. (2013). Spectrum of Bacterial Colonization Associated with Urothelial Cells from Patients with Chronic Lower Urinary Tract Symptoms. J. Clin. Microbiol..

[66] Jiménez-Guerra G., Lara-Oya A., Martínez-Egea I., Navarro-Marí J.M., Gutiérrez-Fernández J. (2018). Urinary tract infection by *Aerococcus sanguinicola*. An emerging opportunistic pathogen. Rev. Clín. Esp..

[67] Pereira-Pérez E., Aparicio-Gómez J.A., Gómez-Camarasa C., Gutiérrez-Fernández J. (2019). A study of urinary tract infections by *Streptococcus gallolyticus* ssp. *pasteurianus*. Rev. Esp. Quimioter..

[68] Ruiz-Pino M., Foronda-García-Hidalgo C., Alarcón-Blanco P., Gutiérrez-Fernández J. (2019). Male genitourinary infections by *Corynebacterium glucuronolyticum*. A review and clinical experience. Rev. Esp. Quimioter..

[69] Heras-Cañas V., Ros L., Sorlózano A., Gutiérrez-Soto B., Navarro-Marí J.M., Gutiérrez-Fernández J. (2015). Especies de levaduras aisladas en muestras de orina en un hospital regional de España. Rev. Argent. Microbiol..

[70] Moustafa A., Li W., Singh H., Moncera K.J., Torralba M.G., Yu Y., Manuel O., Biggs W., Venter J.C., Nelson K.E. (2018). Microbial metagenome of urinary tract infection. Sci. Rep..

[71] Hiergeist A., Gessner A. (2017). Clinical implications of the microbiome in urinary tract diseases. Curr. Opin. Urol..

[72] Tolani M.A., Suleiman A., Awaisu M., Abdulaziz M.M., Lawal A.T., Bello A. (2020). Acute urinary tract infection in patients with underlying benign prostatic hyperplasia and prostate cancer. Pan Afr. Med. J..

[73] Heyns C.F. (2012). Urinary tract infection associated with conditions causing urinary tract obstruction and stasis, excluding urolithiasis and neuropathic bladder. World J. Urol..

[74] Fan C.-Y., Huang W.-Y., Lin K.-T., Lin C.-S., Chao H.-L., Yang J.-F., Lin C.-L., Kao C.-H. (2017). Lower Urinary Tract Infection and Subsequent Risk of Prostate Cancer: A Nationwide Population-Based Cohort Study. PLoS ONE.

[75] Lin J.L., Donegan S.P., Heeren T.C., Greenberg M., Flaherty E.E., Haivanis R., Su X., Dean D., Newhall W.J., Knapp J.S. (1998). Transmission of *Chlamydia trachomatis* and *Neisseria gonorrhoeae* among Men with Urethritis and Their Female Sex Partners. J. Infect. Dis..

[76] Marcus J.L., Kohn R.P., Barry P.M., Philip S.S., Bernstein K.T. (2011). *Chlamydia trachomatis* and *Neisseria gonorrhoeae* Transmission From the Female Oropharynx to the Male Urethra. Sex. Transm. Dis..

[77] Huang W.-Y., Hayes R., Pfeiffer R., Viscidi R.P., Lee F.K., Wang Y.F., Reding D., Whitby D., Papp J.R., Rabkin C.S. (2008). Sexually Transmissible Infections and Prostate Cancer Risk. Cancer Epidemiol. Biomark. Prev..

[78] Sutcliffe S., Zenilman J.M., Ghanem K.G., Jadack R.A., Sokoll L.J., Elliott D.J., Nelson W.G., De Marzo A.M., Cole S.R., Isaacs W.B. (2006). Sexually Transmitted Infections and Prostatic Inflammation/Cell Damage as Measured by Serum Prostate Specific Antigen Concentration. J. Urol..

[79] Hurst R., Meader E., Gihawi A., Rallapalli G., Clark J., Kay G.L., Webb M., Manley K., Curley H., Walker H. (2022). Microbiomes of Urine and the Prostate Are Linked to Human Prostate Cancer Risk Groups. Eur. Urol. Oncol..

[80] Liss M., Lee J., White J., Johnson-Pais T., Lai Z., Troyer D., Leach R., Wickes B. (2022). MP37-08 urinary microbiome as a biomarker for prostate cancer. J. Urol..

[81] Garbas K., Zapała P., Zapała Ł., Radziszewski P. (2021). The Role of Microbial Factors in Prostate Cancer Development—An Up-to-Date Review. J. Clin. Med..

[82] Shrestha E., White J.R., Yu S.-H., Kulac I., Ertunc O., De Marzo A.M., Yegnasubramanian S., Mangold L.A., Partin A.W., Sfanos K.S. (2018). Profiling the Urinary Microbiome in Men with Positive versus Negative Biopsies for Prostate Cancer. J. Urol..

[83] Alanee S., El-Zawahry A., Dynda D., Dabaja A., McVary K., Karr M., Braundmeier-Fleming A. (2019). A prospective study to examine the association of the urinary and fecal microbiota with prostate cancer diagnosis after transrectal biopsy of the prostate using 16sRNA gene analysis. Prostate.

[84] Yu H., Meng H., Zhou F., Ni X., Shen S., Das U.N. (2015). Urinary microbiota in patients with prostate cancer and benign prostatic hyperplasia. Arch. Med. Sci..

[85] Eisenhofer R., Minich J.J., Marotz C., Cooper A., Knight R., Weyrich L.S. (2019). Contamination in Low Microbial Biomass Microbiome Studies: Issues and Recommendations. Trends Microbiol..

[86] Massari F., Mollica V., Di Nunno V., Gatto L., Santoni M., Scarpelli M., Cimadamore A., Lopez-Beltran A., Cheng L., Battelli N. (2019). The Human Microbiota and Prostate Cancer: Friend or Foe?. Cancers.

[87] Sfanos K.S., Isaacs W.B., De Marzo A.M. (2013). Infections and inflammation in prostate cancer. Am. J. Clin. Exp. Urol..

[88] Ragnarsdóttir B., Lutay N., Grönberg-Hernandez J., Köves B., Svanborg C. (2011). Genetics of innate immunity and UTI susceptibility. Nat. Rev. Urol..

[89] Miyake M., Ohnishi K., Hori S., Nakano A., Nakano R., Yano H., Ohnishi S., Owari T., Morizawa Y., Itami Y. (2019). *Mycoplasma genitalium* Infection and Chronic Inflammation in Human Prostate Cancer: Detection Using Prostatectomy and Needle Biopsy Specimens. Cells.

[90] Banerjee S., Alwine J.C., Wei Z., Tian T., Shih N., Sperling C., Guzzo T., Feldman M.D., Robertson E.S. (2019). Microbiome signatures in prostate cancer. Carcinogenesis.

[91] Samanta M., Harkins L., Klemm K., Britt W.J., Cobbs C.S. (2003). High Prevalence of Human Cytomegalovirus in Prostatic Intraepithelial Neoplasia and Prostatic Carcinoma. J. Urol..

[92] Martinez-Fierro M.L., Leach R.J., Gomez-Guerra L.S., Garza-Guajardo R., Johnson-Pais T., Beuten J., Morales-Rodriguez I.B., Hernandez-Ordoñez M.A., Calderon-Cardenas G., Ortiz-Lopez R. (2010). Identification of viral infections in the prostate and evaluation of their association with cancer. BMC Cancer.

[93] Zambrano A., Kalantari M., Simoneau A., Jensen J.L., Villarreal L.P. (2002). Detection of human polyomaviruses and papillomaviruses in prostatic tissue reveals the prostate as a habitat for multiple viral infections. Prostate.

[94] Feng Y., Ramnarine V.R., Bell R., Volik S., Davicioni E., Hayes V.M., Ren S., Collins C.C. (2019). Metagenomic and metatranscriptomic analysis of human prostate microbiota from patients with prostate cancer. BMC Genom..

[95] Liang W., Ferrara N. (2016). The Complex Role of Neutrophils in Tumor Angiogenesis and Metastasis. Cancer Immunol. Res..

[96] Yang L., Pang Y., Moses H.L. (2010). TGF-beta and immune cells: An important regulatory axis in the tumor microenvironment and progression. Trends Immunol..

[97] Salachan P.V., Rasmussen M., Fredsøe J., Ulhøi B., Borre M., Sørensen K.D. (2022). Microbiota of the prostate tumor environment investigated by whole-transcriptome profiling. Genome Med..

[98] Han I.-H., Song H.-O., Ryu J.-S. (2020). IL-6 produced by prostate epithelial cells stimulated with *Trichomonas vaginalis* promotes proliferation of prostate cancer cells by inducing M2 polarization of THP-1-derived macrophages. PLoS Negl. Trop. Dis..

[99] Kushwaha B., Devi A., Maikhuri J.P., Rajender S., Gupta G. (2021). Inflammation driven tumor-like signaling in prostatic epithelial cells by sexually transmitted *Trichomonas vaginalis*. Int. J. Urol..

[100] Ma J., Gnanasekar A., Lee A., Li W.T., Haas M., Wang-Rodriguez J., Chang E.Y., Rajasekaran M., Ongkeko W.M. (2020). Influence of Intratumor Microbiome on Clinical Outcome and Immune Processes in Prostate Cancer. Cancers.

[101] Zhang Q., Yu N., Lee C. (2014). Mysteries of TGF-Î^2^ Paradox in Benign and Malignant Cells. Front. Oncol..

[102] Menzies B.E. (2003). The role of fibronectin binding proteins in the pathogenesis of *Staphylococcus aureus* infections. Curr. Opin. Infect. Dis..

[103] Kadowaki T., Nakayama K., Yoshimura F., Okamoto K., Abe N., Yamamoto K. (1998). Arg-gingipain Acts as a Major Processing Enzyme for Various Cell Surface Proteins in *Porphyromonas gingivalis*. J. Biol. Chem..

[104] Joshipura K.J., Rimm E.B., Douglass C.W., Trichopoulos D., Ascherio A., Willett W.C. (1996). Poor Oral Health and Coronary Heart Disease. J. Dent. Res..

[105] Michaud D.S., Izard J., Wilhelm-Benartzi C.S., You D.-H., Grote V.A., Tjønneland A., Dahm C.C., Overvad K., Jenab M., Fedirko V. (2013). Plasma antibodies to oral bacteria and risk of pancreatic cancer in a large European prospective cohort study. Gut.

[106] Famili P., Cauley J.A., Greenspan S.L. (2007). The Effect of Androgen Deprivation Therapy on Periodontal Disease in Men With Prostate Cancer. J. Urol..

[107] Joshi N., Bissada N.F., Bodner D., MacLennan G.T., Narendran S., Jurevic R., Skillicorn R. (2010). Association Between Periodontal Disease and Prostate-Specific Antigen Levels in Chronic Prostatitis Patients. J. Periodontol..

[108] Noack B., Genco R.J., Trevisan M., Grossi S., Zambon J.J., Nardin E. (2001). De Periodontal Infections Contribute to Elevated Systemic C-Reactive Protein Level. J. Periodontol..

[109] Corbella S., Veronesi P., Galimberti V., Weinstein R., Del Fabbro M., Francetti L. (2018). Is periodontitis a risk indicator for cancer? A meta-analysis. PLoS ONE.

[110] Nabil F., Bissada N.A. (2015). Periodontal Treatment Improves Prostate Symptoms and Lowers Serum PSA in Men with High PSA and Chronic Periodontitis. Dentistry.

[111] Boyapati R., Swarna C., Devulapalli N., Sanivarapu S., Katuri K., Kolaparthy L. (2018). Unveiling the link between prostatitis and periodontitis. Contemp. Clin. Dent..

[112] Huang Y., Michaud D.S., Lu J., Carter H.B., Platz E.A. (2019). The association between clinically determined periodontal disease and prostate-specific antigen concentration in men without prostate cancer: The 2009–2010 National Health and Nutrition Examination Survey. Cancer Causes Control.

[113] Kruck S., Hennenlotter J., Amend B., Geiger M., Filipova E., Stuhler V., Schubert T., Todenhofer T., Rausch S., Huettig F. (2017). Chronic Periodontitis Does Not Impact Serum Levels of Prostate-specific Antigen. Anticancer Res..

[114] Cohen R.J., Shannon B.A., McNeal J.E., Shannon T., Garrett K.L. (2005). *Proprionibacterium acnes* associated with inflammation in radical prostatectomy specimens: A possiblew link to cancer evolution?. J. Urol..

[115] Alexeyev O.A., Marklund I., Shannon B., Golovleva I., Olsson J., Andersson C., Eriksson I., Cohen R., Elgh F. (2007). Direct Visualization of *Propionibacterium acnes* in Prostate Tissue by Multicolor Fluorescent In Situ Hybridization Assay. J. Clin. Microbiol..

[116] Drott J.B., Alexeyev O., Bergström P., Elgh F., Olsson J. (2010). *Propionibacterium acnes* infection induces upregulation of inflammatory genes and cytokine secretion in prostate epithelial cells. BMC Microbiol..

[117] Fassi Fehri L., Mak T.N., Laube B., Brinkmann V., Ogilvie L.A., Mollenkopf H., Lein M., Schmidt T., Meyer T.F., Brüggemann H. (2011). Prevalence of *Propionibacterium acnes* in diseased prostates and its inflammatory and transforming activity on prostate epithelial cells. Int. J. Med. Microbiol..

